# Sex modifies the association between urinary albumin-to-creatinine ratio and diabetes among adults in the United States (NHANES 2011–2018)

**DOI:** 10.1186/s13293-022-00462-y

**Published:** 2022-09-30

**Authors:** Yumeng Shi, Huan Hu, Zuxiang Wu, Ji Wu, Zhiqiang Chen, Ping Li

**Affiliations:** Department of Cardiovascular Medicine, The Second Affiliated Hospital of Nanchang University, No. 1 Minde Road, Nanchang, 330006 Jiangxi China

**Keywords:** Urinary albumin-to-creatinine ratio, Diabetes, Sex differences, Male

## Abstract

**Background:**

Studies on the association between urinary albumin-to-creatinine ratio (uACR) and diabetes are limited. We aimed to examine the association between uACR and diabetes among adults in the United States, with particular interest in sex differences.

**Methods:**

Overall, 5307 participants were included in this study. The exposure variable was uACR, where uACR = urine albumin/urine creatinine. The primary outcome of this study was diabetes, defined as self-reported physician diagnosis of diabetes, fasting plasma glucose concentration ≥ 7.0 mmol/L, or use of glucose-lowering drugs.

**Results:**

The average age of the participants in this study was 46.37 ± 17.38 years, 818 (15.41%) had diabetes and the median uACR was 7 mg/g (interquartile range, 4–12 mg/g). There was a significant positive association between uACR and diabetes (per natural log [uACR] increment: OR, 1.81; 95% CI 1.39–2.34). A multivariate logistic regression model demonstrated that per unit increment in LguACR, the diabetes prevalence increased 2.26-fold among male participants (OR 2.26, 95% CI 1.59–3.21). However, in female participants, we observed that uACR was not related to the prevalence of diabetes (odds ratio [OR], 1.28; 95% CI 0.82–2.01). Our findings showed that there was an interaction between sex and uACR (*P* for interaction = 0.049).

**Conclusions:**

A higher uACR is significantly associated with an increased prevalence of diabetes, and sex can modify the relationship between them.

## Highlights

This study evaluated the relationship between urinary albumin-to-creatinine ratio (uACR) and diabetes among adults in the United States of American for the first time, and found that a positive correlation between uACR and diabetes only existed among male participants but not in female participants.

## Introduction

Diabetes is a metabolic disease that is characterized by chronic hyperglycemia and failure of pancreatic β-cells through different mechanisms [1]. Diabetes is a major global public health problem, affecting more than 34 million adults in the United States. Diabetes significantly increases the risk of cardiovascular events, microvascular diseases, and premature death [2]. The risk of cardiovascular and cerebrovascular diseases, dementia, and some malignant tumors is higher in patients with diabetes than in those without diabetes [3]. Furthermore, in the United States, compared to non-diabetic patients, diabetic patients pay higher medical expenses; moreover, diabetes increases indirect costs owing to increased absenteeism, reduced work productivity, disease-related disability, and premature death [4]. In addition to controlling the currently known risk factors related to diabetes, we also need to pay attention to the role of other biomarkers in the occurrence and development of diabetes.

Urinary albumin-to-creatinine ratio (uACR) is a well-known marker of glomerular injury and is, therefore, an important diagnostic marker of chronic kidney disease (CKD) [5]. As a risk factor for CKD [6, 7], it is routinely recommended to evaluate the annual uACR level in the clinical management of diabetic patients to monitor the occurrence and development of diabetic nephropathy (DN) [8, 9]. However, due to the adverse effects of diabetes on the kidneys, the uACR level in this population is high. Therefore, it is unclear whether we need to intervene when individuals have a mild rise in uACR within the normal range, and whether a mild rise will lead to adverse events. It is very important to study the relationship between normal uACR and chronic diseases, such as diabetes.

Therefore, we used the National Health and Nutrition Examination Survey (NHANES) data from 2011 to 2018 to study the relationship between uACR and diabetes among adults in the United States. We also examined possible effect modifiers between uACR and diabetes.

## Methods

### Study design and population

The National Health and Nutrition Examination Survey (NHANES) is a program of studies designed to assess the health and nutritional status of adults and children in the United States. In the current study, we employed data from four NHANES cycles (2011–2012, 2013–2014, 2015–2016, and 2017–2018) to investigate the association between uACR and T2MD among male and female individuals. The ethics review committee of the National Center for Health Statistics (NCHS) approved the NHANES' research plan. All participants provided written informed consent. Further details can be obtained from the website www.cdc.gov/nchs/nhanes/irba98.htm. In the NHANES study from 2011 to 2018, 10,654 participants aged > 18 years with complete laboratory urine data and diabetes data were included. Demographic, examination, laboratory, and questionnaire data were collected. Individuals with missing values for the covariates (see “Potential covariates” section; *n* = 5347) were excluded. Finally, 5307 subjects were included in our study.

### Definition of uACR and diabetes

Professionally trained researchers obtained 5 mL of self-collected urine from each participant, and sent frozen urine samples (≤ − 20 °C) to the laboratory. Specimen stability was demonstrated at 5 °C and temperatures less than or equal to − 20 °C. Urine albumin was measured using solid-phase fluorescent immunoassay, and urine creatinine was measured using the Roche/Hitachi Modular P Chemistry Analyzer in 2011 and Roche/Hitachi Cobas 6000 chemistry analyzer in 2013. Urine albumin and creatinine levels were standardized and calibrated with the gold standard method according to the recommendations of the National Health and Nutrition Examination Survey. The exposure variable was uACR, where uACR = urine albumin/urine creatinine.

The primary outcome of this study was diabetes. Diabetes as diagnosed according to the standards of the American Diabetes Association [10] and participants’ self-reported questionnaires. diabetes was diagnosed. Each of the following conditions was diagnosed as diabetes: fasting plasma glucose ≥ 7 mmol/L, self-reported physician diagnosis of diabetes, or current use of diabetes medication to lower blood glucose level.

### Potential covariates

Covariates were included as potential confounders in the final multivariate logistic regression models if the estimates of uACR for diabetes changed by more than 10% [11], or were known as traditional risk factors for diabetes. The following variables were used to construct the fully adjusted model: continuous variables included age, poverty income ratio, body mass index (BMI, kg/m^2^), systolic blood pressure (SBP, mmHg), diastolic blood pressure (DBP, mmHg), alcohol intake (drinks per day), fasting blood glucose (FBG, mg/dL), triglycerides (TG, mg/dL), total cholesterol (TC, mg/dL), high-density lipoprotein cholesterol (HDL-C, mg/dL), low-density lipoprotein cholesterol (LDL-C, mg/dL), and estimated glomerular filtration rate (eGFR, mL/min/1.73 m2). Categorical variables included sex (male or female), race (non-Hispanic white, non-Hispanic black, Mexican American, other Hispanic, or others), smoking status (never, former, or current), hypertension, antihypertensive drugs, and lipoprotein-lowering drugs. Furthermore, eGFR was calculated using the Chronic Kidney Disease (CKD) Epidemiology Collaboration equation [12]. Hypertension was defined as a self-reported physician diagnosis of hypertension, SBP ≥ 140 mmHg and/or DBP ≥ 90 mmHg, or the use of antihypertensive drugs [13].

### Statistical analysis

Continuous variables were compared using one-way analysis of one-way ANOVA among the different groups. Categorical variables were compared using the chi-squared test or Fisher’s exact test among the different groups. Continuous variables are summarized as mean ± standard deviation, while categorical variables are expressed as counts (percentage). Because of the skewed distribution of uACR in this study, Log10 conversion (Lg uACR) was carried out in the data analysis. Multivariate logistic regression analysis was used to evaluate the prevalence of diabetes based on the uACR (continuous and categorical variables). Three models were constructed for regression analysis: Model 1 was adjusted for none and Model 2 for age, sex (only for the overall population), race, poverty income ratio, BMI, SBP, DBP, current smoking, alcohol intake, FPG, TG, TC, HDL, and LDL. In model 3, eGFR, antihypertensive drugs, and lipoprotein-lowering drugs were considered, in addition to model 2 adjustments. A generalized additive model and fitted smoothing curve (penalized spline method) were used to characterize the shape of the relationship between uACR and diabetes. Furthermore, possible modifications of the association between uACR and diabetes were assessed for the following variables: age (< 65 vs. ≥ 65 years), race (non-Hispanic white vs. non-Hispanic black vs. Mexican American vs. other Hispanic vs. other races), BMI (< 24 vs. ≥ 24 kg/m^2^), smoking status (never vs. former vs. current), hypertension (yes vs. no), and eGFR (< 60 vs. ≥ 60 mL/min/1.73 m^2^).

Statistical analyses were conducted using R, version 4.2.0 (R Foundation) and Empower Stats (http://www.empowerstats.com, X&Y Solutions, Inc., Boston, MA). A two‐tailed *P* value < 0.05 was considered statistically significant.

## Results

### Study participants and baseline characteristics

Overall, 5307 participants (average age, 46.37 ± 17.38 years) were included in this study. Among the participants, 818 (15.41%) were diabetic patients. The median uACR of the participants was 7 mg/g (interquartile range, 4–12 mg/g). The average age and median uACR of the 2837 male participants were 46.93 ± 17.47 years and 6 mg/g (interquartile range, 4–11 mg/g), respectively. The average age and median uACR of the 2470 female participants were 45.73 ± 17.26 years and 8 mg/g (interquartile range, 5–14 mg/g), respectively. The prevalence of diabetes in male and female participants was 484 (17.06%) and 334 (13.52%), respectively.

The demographics of the study population according to tertiles of uACR among male and female participants from the NHANES (2011–2018) are shown in Table 1. Male participants with a medium to high uACR were generally older, current smokers, and non-Hispanic white; had higher values for BMI, SBP, DBP, FPG, TC, diabetes, hypertension, antihypertensive drugs, and lipoprotein-lowering drugs; and had lower levels of poverty income ratio, LDL, and eGFR, compared to those with very low uACR. Female participants with medium to high uACR tended to be older current smokers and non-Hispanic white, had higher levels of SBP, DBP, FPG, TC, diabetes, hypertension, antihypertensive drugs, and lipoprotein-lowering drugs, and lower values of poverty income ratio and eGFR, compared to those with very low uACR.

**Table 1:** Baseline characteristics of study participants

Characteristics^a^	Males			*P* value^b^	Females			*P* value^c^
	uACR (mg/g)^d^ tertiles				uACR (mg/g)^d^ tertiles			
	Tertiles 1	Tertile 2	Tertile 3		Tertiles 1	Tertile 2	Tertile 3	
uACR range	< 4	4–8	≥ 8		< 6	6–11	≥ 11	
*N*	946	945	946		823	823	824	
Age, years	40.70 ± 15.42	45.79 ± 17.06	54.30 ± 17.10	< 0.001	41.77 ± 15.27	45.82 ± 17.11	49.60 ± 18.37	< 0.001
Race				0.011				0.002
Non-Hispanic White, %	390 (41.23%)	419 (44.34%)	388 (41.01%)		335 (40.70%)	362 (43.99%)	356 (43.20%)	
Non-Hispanic Black, %	191 (20.19%)	152 (16.08%)	202 (21.35%)		221 (26.85%)	151 (18.35%)	179 (21.72%)	
Mexican American, %	119 (12.58%)	127 (13.44%)	146 (15.43%)		85 (10.33%)	112 (13.61%)	96 (11.65%)	
Other Hispanic, %	86 (9.09%)	91 (9.63%)	92 (9.73%)		72 (8.75%)	81 (9.84%)	96 (11.65%)	
Other races, %	160 (16.91%)	156 (16.51%)	118 (12.47%)		110 (13.37%)	117 (14.22%)	97 (11.77%)	
BMI, kg/m^2^	28.20 ± 5.37	28.00 ± 5.90	29.46 ± 6.98	< 0.001	29.72 ± 7.83	29.38 ± 7.63	29.51 ± 8.42	0.690
SBP, mmHg	119.30 ± 12.97	123.32 ± 14.47	131.64 ± 19.40	< 0.001	114.89 ± 13.79	119.09 ± 16.52	125.64 ± 21.49	< 0.001
DBP, mmHg	70.26 ± 10.78	71.35 ± 11.81	72.87 ± 13.04	< 0.001	67.70 ± 9.81	69.14 ± 10.96	70.07 ± 12.50	< 0.001
Poverty income ratio	2.70 ± 1.67	2.78 ± 1.69	2.52 ± 1.59	0.002	2.77 ± 1.69	2.68 ± 1.66	2.49 ± 1.62	0.002
Current smoking, %	221 (23.36%)	234 (24.76%)	271 (28.65%)	< 0.001	149 (18.10%)	135 (16.40%)	172 (20.87%)	0.026
Alcohol intake, drinks per day	3.12 ± 2.62	3.23 ± 3.63	3.21 ± 2.87	0.699	2.13 ± 1.57	2.04 ± 1.49	2.09 ± 1.74	0.483
FPG, mg/dL	102.35 ± 18.59	106.27 ± 23.05	123.83 ± 48.97	< 0.001	100.09 ± 23.55	102.07 ± 22.94	112.78 ± 42.19	< 0.001
TC, mg/dL	112.42 ± 67.91	117.22 ± 68.26	126.29 ± 72.45	< 0.001	93.25 ± 55.03	99.22 ± 56.53	107.69 ± 64.27	< 0.001
TG, mg/dL	184.66 ± 38.94	187.17 ± 38.39	184.36 ± 42.18	0.244	189.70 ± 39.42	190.85 ± 38.36	193.38 ± 42.29	0.163
HDL-C, mg/dL	49.48 ± 12.75	50.38 ± 14.30	50.15 ± 15.71	0.363	60.25 ± 16.12	60.17 ± 17.24	60.03 ± 18.59	0.967
LDL-C, mg/dL	112.69 ± 33.89	113.34 ± 34.30	108.96 ± 36.98	0.014	110.81 ± 34.41	110.84 ± 32.77	111.80 ± 36.36	0.803
eGFR, mL/min/1.73 m^2^	97.93 ± 18.30	98.47 ± 20.08	90.76 ± 24.78	< 0.001	100.91 ± 20.92	100.80 ± 22.25	97.41 ± 24.97	0.002
Diabetes	62 (6.55%)	116 (12.28%)	306 (32.35%)	< 0.001	66 (8.02%)	93 (11.30%)	175 (21.24%)	< 0.001
Hypertension	247 (26.11%)	341 (36.08%)	563 (59.51%)	< 0.001	215 (26.12%)	293 (35.60%)	406 (49.27%)	< 0.001
Antihypertensive drugs	26 (2.75%)	45 (4.76%)	70 (7.40%)	< 0.001	33 (4.01%)	54 (6.56%)	57 (6.92%)	< 0.001
Lipoprotein-lowering drugs	120 (12.68%)	167 (17.67%)	296 (31.29%)	< 0.001	85 (10.33%)	135 (16.40%)	180 (21.84%)	< 0.001

*BMI* body mass index, *SBP* systolic blood pressure, *DBP* diastolic blood pressure, *FPG* fasting plasma glucose, *TC* total cholesterol, *TG* triglycerides, *LDL-C* low-density lipoprotein cholesterol, *eGFR* estimated glomerular filtration rate

^a^Data are presented as number (%) or mean ± standard deviation

^b^Comparisons among uACR tertiles in participants with Male

^c^Comparisons among uACR tertiles in participants with Female

^d^uACR value was log10-transformed

However, no significant differences were found in alcohol intake, TC, and HDL levels in both male and female participants in the three groups. In addition, there was no significant difference between the BMI and LDL levels in female participants among the three groups.

### Associations between uACR and diabetes

**Fig. 1:**
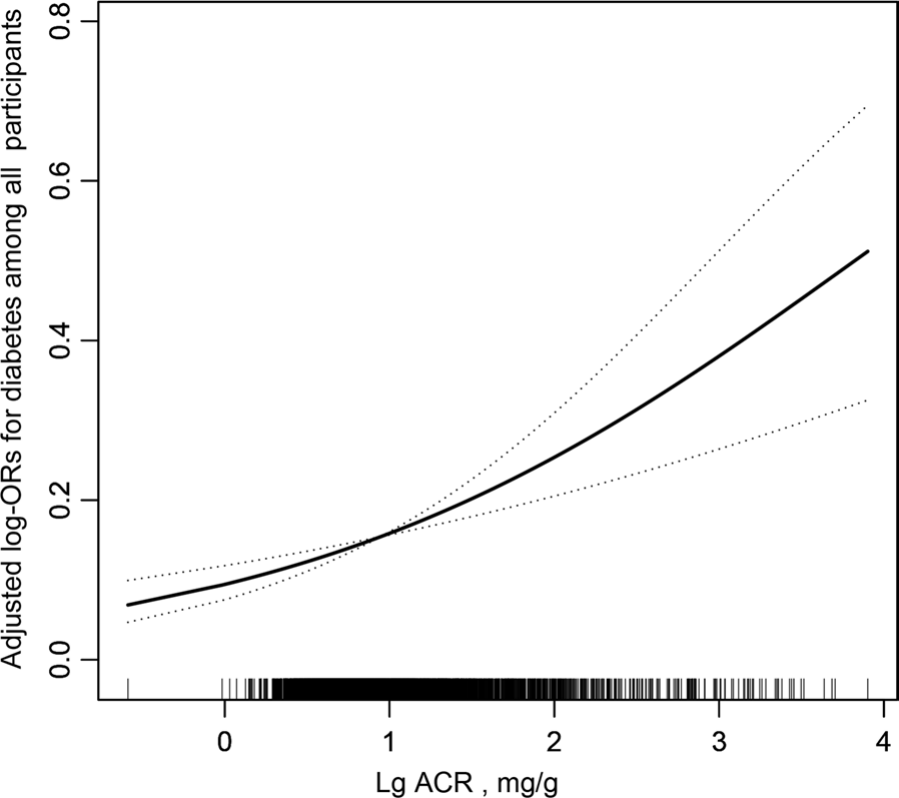
Association between uACR and the prevalence of diabetes. A linear association between uACR and the prevalence of diabetes was found (*P* < 0.05). The solid line and dashed line represent the estimated values and their corresponding 95% confidence interval. Adjustment factors included age, sex, race, poverty income ratio, BMI, SBP, DBP, hypertension, current smoking, alcohol intake, FPG, TG, TC, HDL, LDL, eGFR, antihypertensive drugs, lipoprotein-lowering drugs

**Fig. 2:**
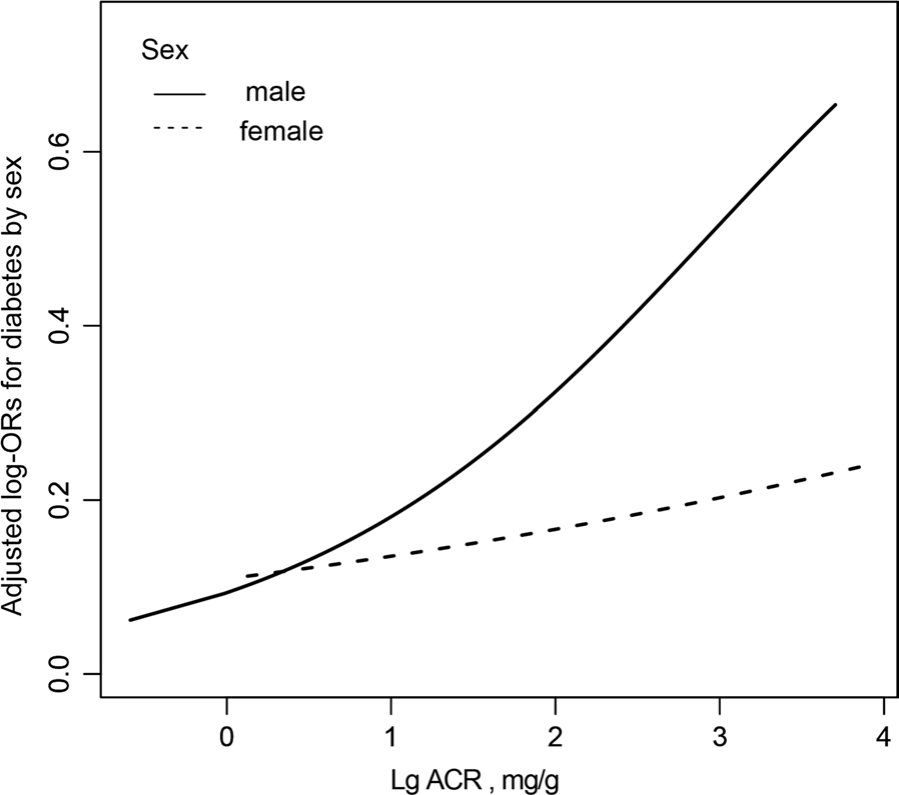
Association between uACR and the prevalence of diabetes by sex. A linear association between TBil and the prevalence of PAD by sex was found (*P* < 0.05). The solid line and dashed line represent the estimated values in male and female, respectively. The adjustment factors included age, race, poverty income ratio, BMI, SBP, DBP, hypertension, current smoking, alcohol intake, FPG, TG, TC, HDL, LDL, eGFR, antihypertensive drugs, lipoprotein-lowering drugs

The results of the multivariate analyses are presented in Table 2. Overall, there was a significant positive association between uACR and diabetes (per natural log [uACR] increment: OR, 1.81; 95% CI 1.39–2.34) (Fig. 1, Table 2). When continuous uACR was converted to tertiles of uACR, we found that compared to those in tertile 1 (uACR < 5, mg/g), the adjusted ORs for participants in tertile 2 (5 ≤ uACR < 10, mg/g) and tertile 3 (uACR ≥ 10, mg/g) were 1.37 (95% CI 0.97–1.94) and 1.82 (95% CI 1.29–2.56) (*P* < 0.001), respectively. The results of the generalized additive model and fitted smoothing curve (penalized spline method) showed a positive association between uACR and the prevalence of diabetes among male but not female participants (Fig. 2). Consistently, a multivariate logistic regression model demonstrated that per unit increment in LguACR, the diabetes prevalence increased 2.26-fold among male participants (OR 2.26, 95% CI 1.59–3.21). However, in female participants, we found that uACR was not related to the prevalence of diabetes (odds ratio [OR], 1.28; 95% CI 0.82–2.01). There was an interaction between sex and uACR (*P* for interaction = 0.049).

**Table 2:** Relative odds of diabetes according to uACR in different models among US adults

uACR, mg/g^†^	Events (%)	Diabetes OR (95% CI), *P* value		
		Model 1	Model 2	Model 3
All participants				
Continuous (Lg uACR)	818 (15.41%)	3.89 (3.36, 4.50), < 0.001	1.90 (1.47, 2.46), < 0.001	1.81 (1.39, 2.34), < 0.001
Tertile 1 (< 5)	119 (6.73%)	Reference	Reference	Reference
Tertile 2 (5–10)	221 (12.49%)	1.98 (1.57, 2.50), < 0.001	1.36 (0.97, 1.91), 0.079	1.37 (0.97, 1.94) 0.077
Tertile 3 (≥ 10)	478 (27.02%)	5.13 (4.15, 6.36), < 0.001	1.86 (1.33, 2.59), < 0.001	1.82 (1.29, 2.56), < 0.001
*P* for trend		< 0.001	< 0.001	< 0.001
Male				
Continuous (Lg uACR)	484 (17.06%)	4.75 (3.91, 5.76), < 0.001	2.43 (1.72, 3.42), < 0.001	2.26 (1.59, 3.21), < 0.001
Tertile 1 (< 4)	62 (6.55%)	Reference	Reference	Reference
Tertile 2 (4–8)	116 (12.28%)	2.00 (1.45, 2.75), < 0.001	1.10 (0.68, 1.79), 0.693	1.11 (0.67, 1.81) 0.691
Tertile 3 (≥ 8)	306 (32.35%)	6.82 (5.09, 9.12), < 0.001	1.95 (1.22, 3.10), 0.005	1.94 (1.20, 3.13) 0.007
*P* for trend		< 0.001	0.002	0.003
Female				
Continuous (Lg uACR)	334 (13.52%)	2.99 (2.36, 3.78), < 0.001	1.23 (0.79, 1.90), 0.359	1.28 (0.82, 2.01), 0.278
Tertile 1 (< 6)	66 (8.02%)	Reference	Reference	Reference
Tertile 2 (6–11)	93 (11.30%)	1.46 (1.05, 2.04), 0.025	0.95 (0.59, 1.53), 0.843	0.96 (0.59, 1.57), 0.881
Tertile 3 (≥ 11)	175 (21.24%)	3.09 (2.29, 4.18), < 0.001	1.07 (0.67, 1.70), 0.784	1.11 (0.68, 1.80), 0.678
*P* for trend		< 0.001	0.751	0.644
*P* value for interaction*		0.003	0.014	0.049

Values are ORs (95% CIs) unless otherwise indicated. uACR, albumin-to-creatinine ratio. ^†^uACR value was log10-transformed

Model 1 was adjusted for none

Model 2 was adjusted for age, sex (only for overall population), race, poverty income ratio, BMI, SBP, DBP, current smoking, alcohol intake, FPG, TG, TC, HDL, LDL

Model 3 was adjusted for age, sex (only for overall population), race, poverty income ratio, BMI, SBP, DBP, hypertension, current smoking, alcohol intake, FPG, TG, TC, HDL, LDL, eGFR, antihypertensive drugs, lipoprotein-lowering drugs

*P value for interaction test: two-way interaction of sex and LguACR (continuous) on diabetes

### Subgroup analyses

**Fig. 3:**
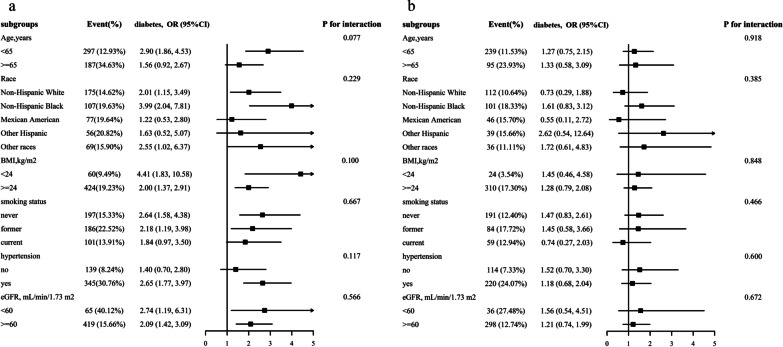
Stratified Analyses by Potential Modifiers of the Association between uACR and the prevalence of diabetes by sex* **a** males; **b** females. *Each subgroup analysis adjusted for age, race, poverty income ratio, BMI, SBP, DBP, hypertension, current smoking, alcohol intake, FPG, TG, TC, HDL, LDL, eGFR, antihypertensive drugs, lipoprotein-lowering drugs. except for the stratifying variable

A stratified analysis was conducted among male and female participants to evaluate the relationship between uACR (continuous variable) and the prevalence diabetes in various subgroups (Fig. 3). In both male (Fig. 3a) and female (Fig. 3b) participants, none of the variables, including age, race, BMI, smoking status, hypertension, and eGFR, significantly modified the association between uACR and the prevalence of diabetes (P for all interactions > 0.05).

## Discussion

This study found an association between uACR and the prevalence of diabetes among adults in the United States. A higher uACR is significantly associated with an increased prevalence of diabetes, and sex can modify the relationship between them. In particular, uACR was positively correlated with the prevalence of diabetes only among male participants; this relationship was not observed in female participants.

Previous studies have not evaluated the relationship between uACR and diabetes, and most have observed a relationship between uACR and its related complications in patients with diabetes. Divya et al. explored the relationship between uACR and diabetic retinopathy (DR) in 272 patients with diabetes. The results showed that the increase in uACR was independently related to the severity of DR in patients with diabetes [14]. In addition, a case–control study involving 180 participants showed that uACR was superior to urinary neutrophil gelatinase-associated lipocalin (NGAL) and urinary Interleukin-18 (IL-18) in predicting diabetic DR [15]. According to the above research, uACR was shown to be closely related to complications in diabetic patients; however, the study population was from a single data set and the sample size was too small.

Based on previous literature, this study is the first to evaluate the relationship between uACR and the prevalence of diabetes among adults in the United States, which fills a gap in this research field. Our large-scale cross-sectional results provide new insights. First, the results of this study showed that there is a significant linear positive correlation between uACR and the prevalence of diabetes; with an increase in uACR levels, the prevalence of diabetes also increases in a stepwise fashion. Second, we found that there was an interaction between sex and uACR, and that sex can modify the relationship between uACR and diabetes (*P* for interaction = 0.049). A higher uACR level was significantly associated with an increased incidence of diabetes among male participants, but not in female participants. The above significant sex differences can be explained by the differences in sex hormones between male and female participants. Studies have shown that estrogen can reduce the risk of insulin resistance and diabetes in women; hence, it has an obvious protective effect in women [16]. However, androgens can increase the risk of insulin resistance (IR) [17, 18], and epidemiological studies have shown that impaired fasting blood glucose (IFG) is more common in men [19, 20]. Therefore, men are more vulnerable to diabetes than women are. Moreover, a recent study showed sex-dependent higher basal levels of creatinine in male individuals than in female individuals, as a distinctive pattern linked to the sex of an individual [21]. Although creatinine can be detected in many tissues, its concentration and distribution are always different. Creatinine levels in the skeletal muscle, heart, and sperm are the highest [22]. Above all, compared with women, men have higher creatinine levels and lower insulin sensitivity; therefore, the positive correlation between uACR and diabetes is more significant in men. Finally, the results of the subgroup analysis showed that a stable positive correlation between uACR and diabetes exists in every male subgroup. In addition, the relationship between them was stable among the female subgroups.

Although the pathophysiological relationship between uACR and diabetes is unclear, it seems reasonable in terms of biology. In an observational study conducted by Utsunomiya et al. [23]. among 752 Japanese participants, an increase in uACR levels was observed to be closely related to insulin resistance (IR). Increased uACR levels indicate that glomerular endothelial injury has led to albumin leakage, which also reflects systemic endothelial injury [24, 25], Damage, including abnormal fibrinolytic and coagulation pathways, microinflammation, and oxidative stress are closely related to the pathological process of insulin resistance. Moreover, insulin resistance is known as the main feature of diabetes [26, 27]. However, more research is needed to confirm our findings and further explore the potential mechanisms underlying them.

## Limitations

Our study has some limitations. First, its cross-sectional nature excludes the inference of causality. Second, owing to the observational nature of this study, although a wide range of confounding factors of prior selection were adjusted other confounding factors still exist. Third, the observed relationship is limited to adults in the United States, which may limit the extrapolation of the results. Considering these limitations, a well-designed prospective cohort trial is required to verify our findings.

## Future directions

This study emphasizes the importance of uACR in the prevalence of diabetes, especially among adult men in the United States. In clinical practice, doctors should closely monitor the uACR of adult men to prevent the occurrence and development of diabetes and related diseases.

## Conclusions

In conclusion, the present study examined whether sex modifies the association between uACR and diabetes among adults in the United States. We found that the positive correlation between uACR and diabetes was more significant among male participants than in female participants.

## Data Availability

Publicly available data sets were analyzed in this study. This data can be found here: https://www.cdc.gov/nchs/nhanes/index.htm.
